# Management of chronic Monteggia lesion with severe radial head deformity using ulnar osteotomy, radial head replacement and ligament repair: A technical note

**DOI:** 10.1051/sicotj/2026059

**Published:** 2026-09-07

**Authors:** Jiuzhou Lu, Erica Kholinne, Kangqi Yang, Yudong Yang, Yiqing Xue, Shulin Li, Jianyuan Jiang, Jianguang Xu

**Affiliations:** 1 Department of Orthopedic Surgery, Huashan Hospital, Fudan University Shanghai PR China; 2 Orthopedics and Traumatology, Gatam Institute, Eka Hospital Tangerang 15321 Indonesia; 3 Department of Surgery, Faculty of Medicine, Universitas Trisakti Jakarta 11440 Indonesia; 4 Department of Hand Surgery, Huashan Hospital, Fudan University Shanghai PR China; 5 Department of Orthopedic Surgery, Xuhui District Central Hospital Shanghai PR China

**Keywords:** Chronic Monteggia lesion, Radial head dislocation, Ulna osteotomy, Elbow arthroplasty, Radial head replacement

## Abstract

Chronic Monteggia lesions are difficult to treat when longstanding radial head dislocation results in severe radial head deformity. In such cases, corrective ulnar osteotomy with or without annular ligament repair may not achieve stable or congruent radiocapitellar reduction. This technical note describes a combined reconstructive technique using corrective ulnar osteotomy, radial head replacement, and annular ligament/lateral ligament complex repair. Through a Boyd approach, the deformed radial head is resected, and the proximal ulna is osteotomized and dorsally angulated to restore radiocapitellar alignment. The resected radial head may be used as an intercalary graft at the osteotomy site. After trial radial head replacement, the capitellum, radial head notch, and proximal ulna are contoured as needed to improve prosthetic congruity. The annular ligament, lateral ulnar collateral ligament remnant, and capsule are repaired with suture anchors to enhance stability. In chronic Monteggia lesions with severe radial head deformity, this combined technique may be useful when isolated ulnar osteotomy is unlikely to restore stable radio-capitellar articulation. A longer follow-up is needed to evaluate durability, functional outcomes, and complications.

## Introduction

Monteggia lesion is characterized by a fracture of the proximal one-third of the ulna combined with dislocation of the radial head [1]. While the dislocation is frequently neglected in pediatric patients, symptoms such as progressive elbow or wrist pain, limited range of motion, elbow instability, or valgus deformity can emerge over time, even decades after the initial injury [2–4].

**Figure 1:**
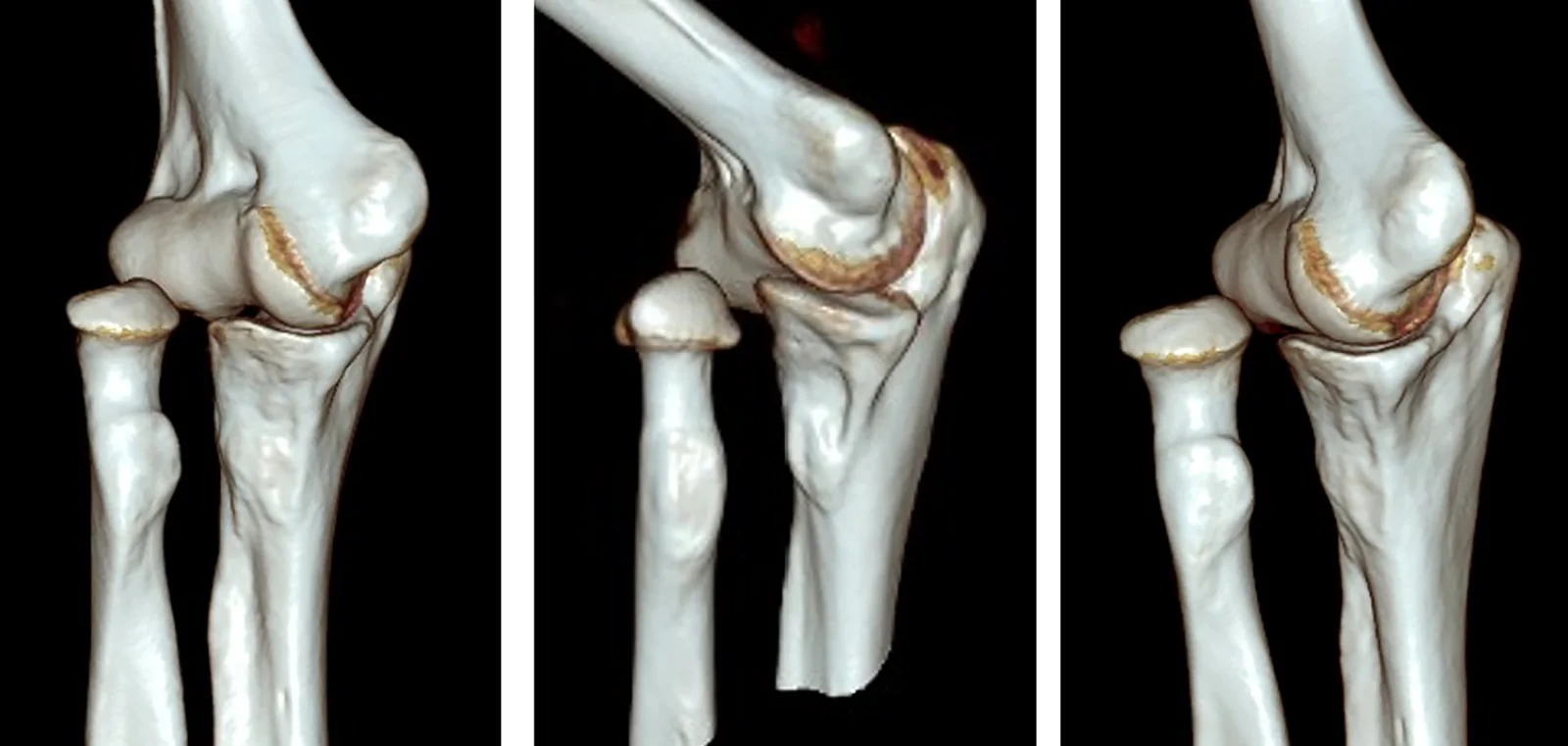
Preoperative computed tomography (CT) images showing chronic radial head dislocation with severe radial head deformity and incongruent radiocapitellar articulation.

The primary objective in the treatment of chronic Monteggia lesion is to restore stable radial head reduction. Conventional surgical strategies generally include corrective ulnar osteotomy and open reduction of the radial head, with or without annular ligament reconstruction or repair [3–7]. These procedures are often effective when the radial head remains sufficiently congruent with the capitellum and radial notch. However, in Monteggia lesions present for over a decade, the radial head may become severely enlarged, flattened, or dysplastic (Figure 1). In these cases, even after adequate ulnar correction, the radial head may remain incongruent with the capitellum and proximal radioulnar joint.

To address this difficult subset of chronic Monteggia lesions, we describe a combined reconstructive technique using corrective ulnar osteotomy, radial head replacement, and annular ligament/lateral ligament complex repair. This approach aims to restore radiocapitellar alignment and improve proximal radioulnar joint congruity in patients whose radial head deformity prevents stable reduction with ulnar osteotomy alone.

## Material and methods

**Inclusion criteria**

- Chronic Monteggia lesion with severe radial head deformity which prevents congruent reduction with isolated ulnar osteotomy.
- Persistent elbow and/or wrist pain, instability, valgus deformity, or mechanical symptoms.
- Skeletally mature patients defined radiographically by the closure of the proximal radial physis.

**Exclusion criteria**

- Active infection.
- Severe capitellar deformity preventing stable prosthetic articulation.
- Nonfunctional elbow or neurologically compromised limb.
- Inability to comply with postoperative rehabilitation.
- Patients with advanced ulnohumeral arthritic changes.

### Preoperative evaluation and planning

Preoperative evaluation should include a detailed clinical and radiographic assessment of the elbow, forearm, and wrist. Clinical examination should document elbow flexion-extension, forearm pronation-supination, valgus alignment, pain location, mechanical symptoms, and subjective instability. Wrist symptoms should also be assessed because chronic radial head dislocation may affect longitudinal forearm stability. Computed tomography (CT) with three-dimensional reconstruction is recommended to evaluate the severity of radial head deformity, capitellar dysplasia, and proximal ulnar deformity.

Preoperative planning should determine the approximate level and direction of ulnar osteotomy, the expected degree of correction, and the feasibility of radial head reconstruction versus replacement.

### Surgical technique

#### Patient positioning and superficial dissection

The patient is placed in the lateral decubitus position with the operative arm supported on a padded arm holder, with the shoulder positioned in approximately 90° of abduction. A pneumatic tourniquet is applied and inflated to 250 mmHg after exsanguination.

**Figure 2:**
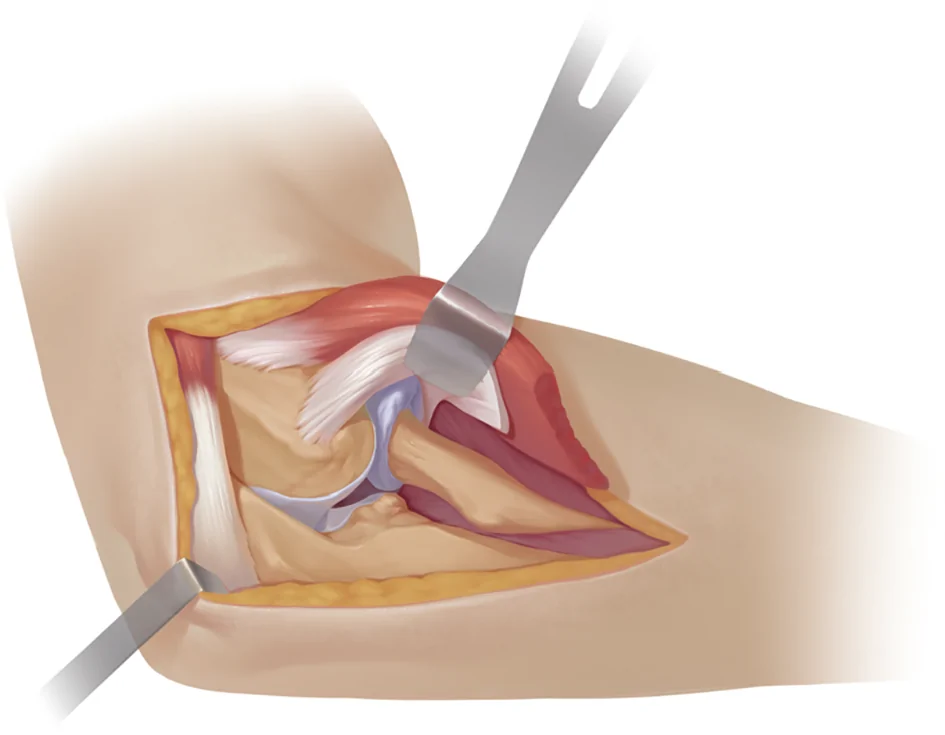
Exposure through the Boyd approach, demonstrating access to the proximal ulna, radial head, radial notch, and proximal radioulnar joint through a single posterolateral incision.

A Boyd approach is used, with a posterior skin incision made from the lateral epicondyle to the lateral border of the olecranon and extended distally for 6–8 cm. The anconeus and extensor muscles are elevated subperiosteally from the ulna. The lateral collateral ligament–annular ligament complex is detached carefully from its ulnar insertion. This allows direct visualization of the radial head, proximal ulna, and proximal radioulnar joint (Figure 2). Scar tissue and interposed fibrous tissue are released sufficiently to allow mobilization of the proximal radius and assessment of radiocapitellar reducibility.

Care should be taken to avoid excessive dissection around the radial neck to minimize the risk of posterior interosseous nerve injury. In cases with coronoid overgrowth or osteophyte formation that limits visualization or reduction, careful trimming should be performed to improve access to the radial head and proximal radioulnar joint.

#### Radial head resection

**Figure 3:**
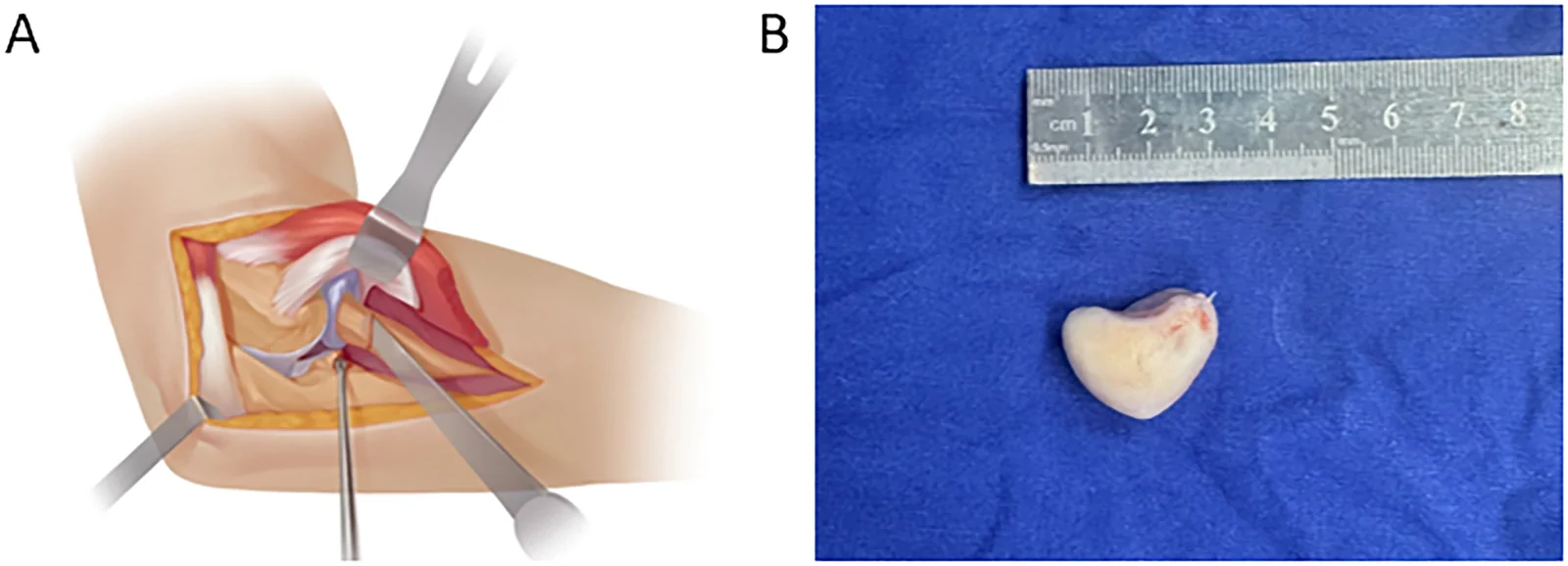
(A) Resection of the deformed radial head perpendicular to the radial neck axis while preserving adequate radial neck bone stock for prosthetic implantation; (B) The resected radial head is prepared as an autologous intercalary bone graft after removal of the articular cartilage.

After adequate exposure, the radial head is resected using an oscillating saw perpendicular to the axis of the radial neck. The resection level is selected to preserve as much radial neck bone stock as possible while allowing adequate canal preparation and prosthetic seating (Figure 3A). Excessive radial neck shortening should be avoided because it may contribute to longitudinal instability, under-stuffing, or altered elbow biomechanics. The Posterior Interosseous Nerve (PIN) should be carefully preserved during radial head resection. The resected radial head is preserved. Its cancellous bone may be contoured for use as an intercalary autograft at the ulnar osteotomy site if additional structural support is required (Figure 3B).

#### Ulnar angulation and osteotomy

**Figure 4:**
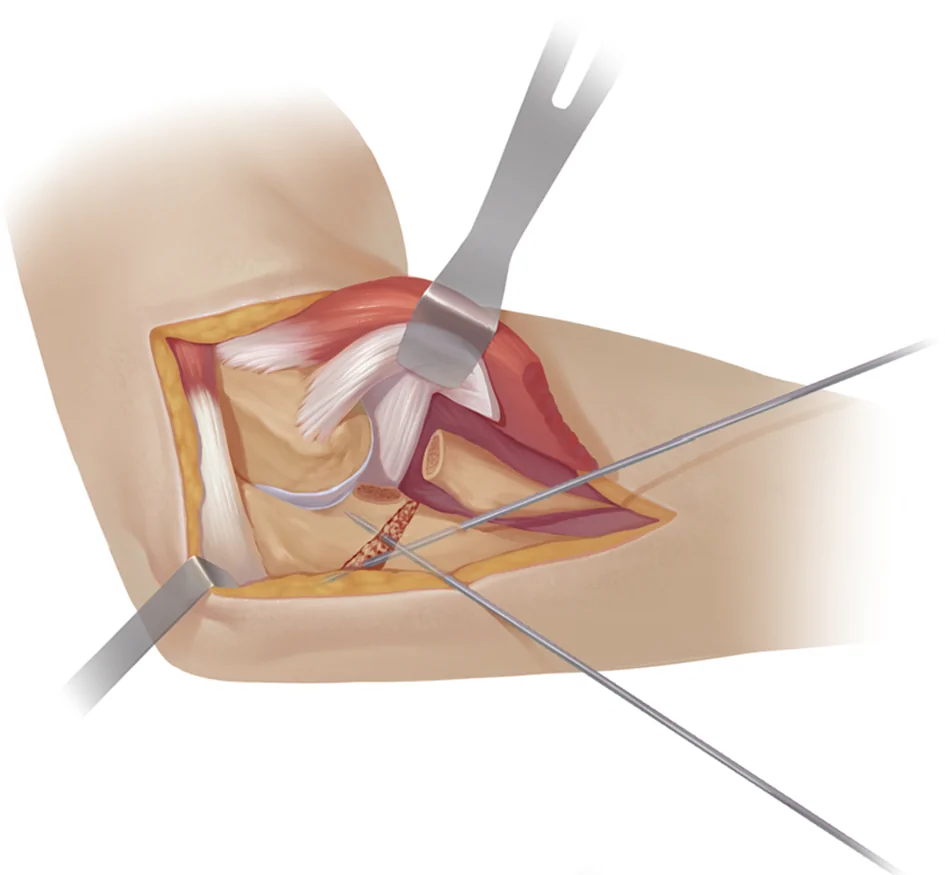
Transverse osteotomy is performed at the proximal ulna at the planned level of correction, with the radial head reduced and osteotomized site angulated dorsally at approximately 30°.

A transverse osteotomy is performed at the proximal ulna at the planned level of correction. With the proximal radius positioned to simulate its intended reduced alignment, the osteotomy is gradually angulated dorsally until concentric radiocapitellar articulation is achieved (Figure 4). In our experience, this typically corresponds to approximately 20-30° of dorsal angulation; however, the final degree of correction should be individualized according to pre-operative 3D-CT reconstruction and intraoperative assessment of the radial head dislocation and ulnar deformity.

The osteotomy is stabilized using a laterally positioned olecranon plate secured with six or seven screws. When appropriate, a central interfragmentary lag screw is added to enhance compression across the osteotomy. If additional structural support is required, the previously harvested radial head autograft is contoured and fitted into the osteotomy gap before being compressed and stabilized as part of the osteotomy construct. Intraoperative fluoroscopy is used to confirm ulnar alignment, osteotomy fixation, and restoration of the radiocapitellar relationship.

#### Radial head replacement

The radial canal is prepared after radial head resection. The entry point is identified at the proximal radial neck, and sequential reaming is performed carefully. In chronic Monteggia lesions, the radial neck may be osteopenic or have thin cortices due to longstanding abnormal loading. Therefore, aggressive reaming and forceful press-fit insertion should be avoided to reduce the risk of radial neck fracture or cortical perforation.

Trial components are used to determine the appropriate radial head diameter, height, and stem size. After trial implantation, the congruity between the prosthetic radial head, capitellum, and proximal ulna is assessed.

In longstanding chronic Monteggia lesions, deformed remodeling of the joint may prevent smooth prosthetic tracking. When impingement or incongruity is identified, controlled contouring of the capitellum and radial aspect of the proximal ulna adjacent to the bicipital tuberosity may be performed using a high-speed burr and/or chisel. Only the minimum amount of bone and residual cartilage necessary to eliminate impingement and restore smooth radiocapitellar tracking should be removed. Excessive contouring should be avoided to preserve the articular surface and minimize the potential risk of degenerative changes.

**Figure 5:**
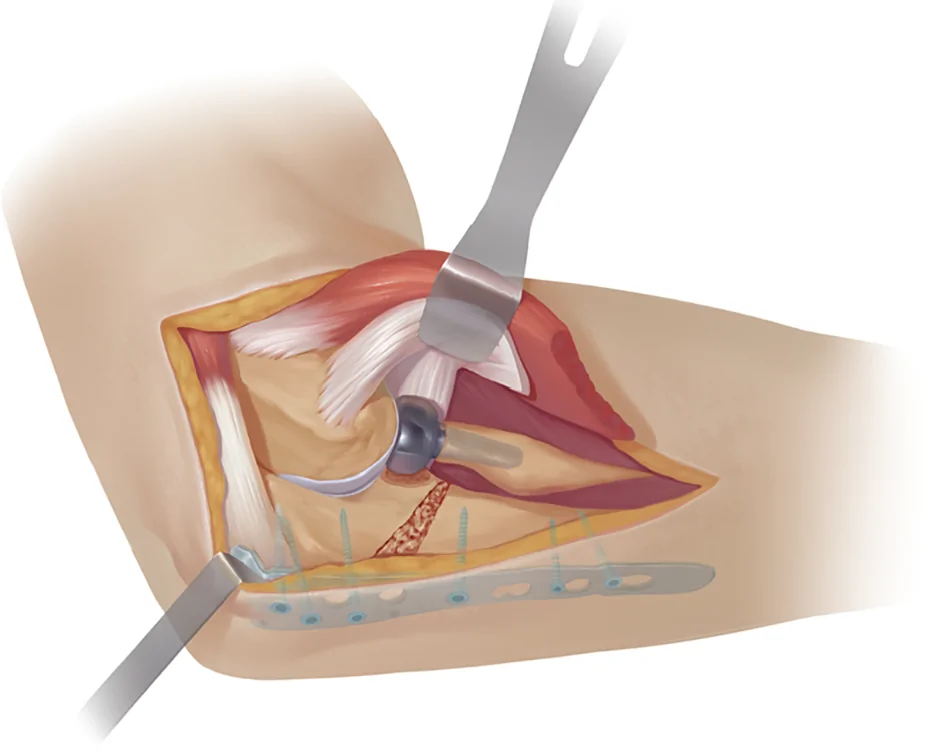
Radial head replacement is performed after assessing implant height, radiocapitellar congruity, elbow stability, and forearm rotation.

**Figure 6:**
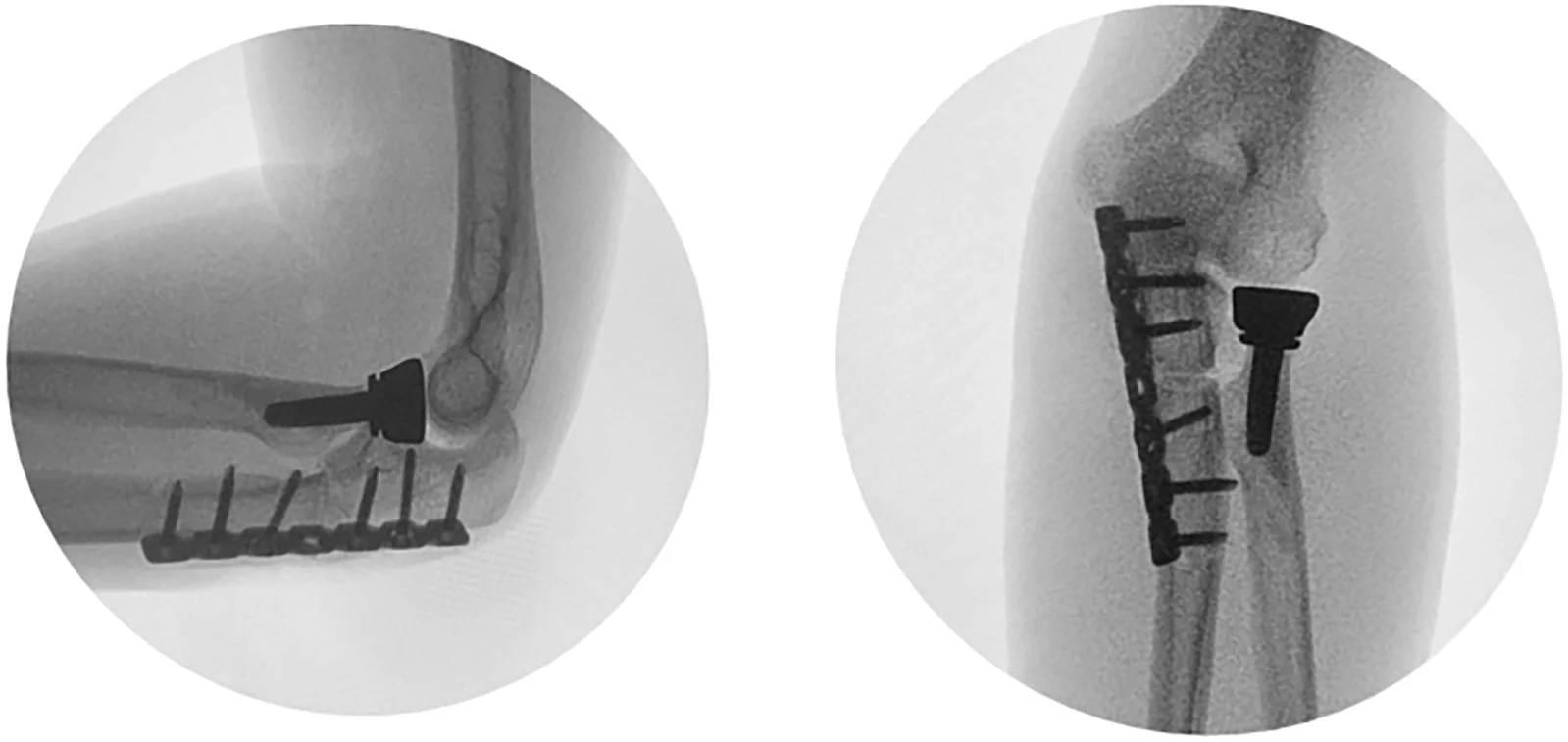
Intraoperative fluoroscopic image confirming ulnar osteotomy fixation, radial head prosthesis position, and restored radiocapitellar alignment.

After satisfactory trialing and contouring, the final radial head prosthesis is implanted into the radial canal (Figure 5). Assessment is then performed through full elbow flexion-extension and forearm rotation. Fluoroscopy is used to confirm implant position (Figure 6).

#### Annular ligament and lateral ligament complex repair

**Figure 7:**
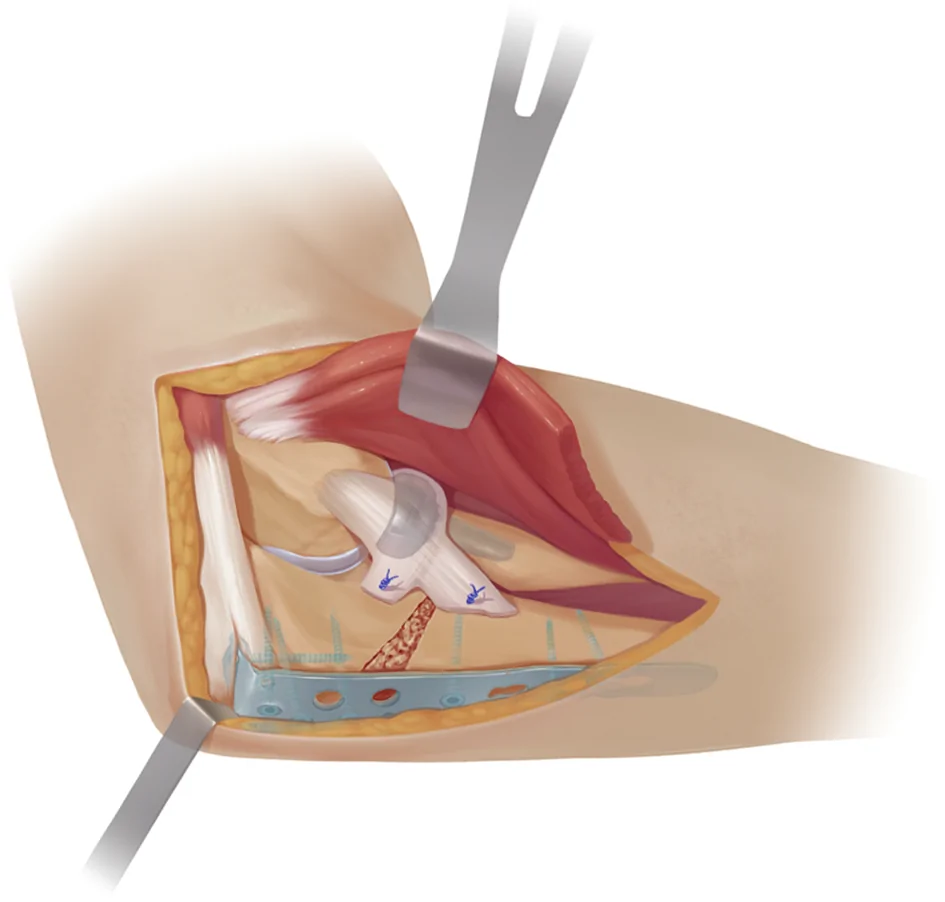
Annular ligament and lateral ligament complex reconstruction using suture anchors to reinforce radial head stability after prosthetic implantation.

After the radial head replacement and ulnar osteotomy fixation, the annular ligament and lateral ligament complex are repaired to improve radial head stability (Figure 7).

The residual annular ligament, lateral ulnar collateral ligament remnant, and joint capsule are mobilized. A standard two 1.8-mm all-suture anchors are placed at the distal insertion area of the lateral ligament complex and annular ligament near the supinator crest and posterior margin of the radial notch. The ligament-capsular complex is repaired securely to restore soft-tissue restraint around the radial head.

The repair is performed with the radial head centered, the elbow flexed approximately 90°, and the forearm in neutral rotation. After repair, stability is assessed through passive elbow flexion-extension and forearm pronation-supination.

#### Closure and dressing

After final irrigation, hemostasis is achieved. The wound is closed in layers according to standard technique. A suction drain is placed to prevent hematoma, and a sterile dressing is applied.

### Postoperative management

Postoperatively, the elbow is immobilized at 90° of flexion with the forearm in neutral rotation, maintained for the first 3 days.

From postoperative day 3 to week 3, assisted mobilization is initiated using a hinged elbow brace under professional supervision. Gentle active-assisted elbow flexion-extension exercises are performed while avoiding excessive varus, valgus, or rotational stress. Forearm rotation is introduced gradually according to pain, stability, and surgeon preference. Exercises are performed several times per day under guidance.

A postoperative CT scan is obtained approximately 1 week after surgery to evaluate radial head prosthesis position, osteotomy alignment, fixation stability, and radiocapitellar/proximal radioulnar congruity. Clinical and radiographic follow-up is performed at 1, 3, and 6 months postoperatively, with additional follow-up as needed.

## Results

**Figure 8:**
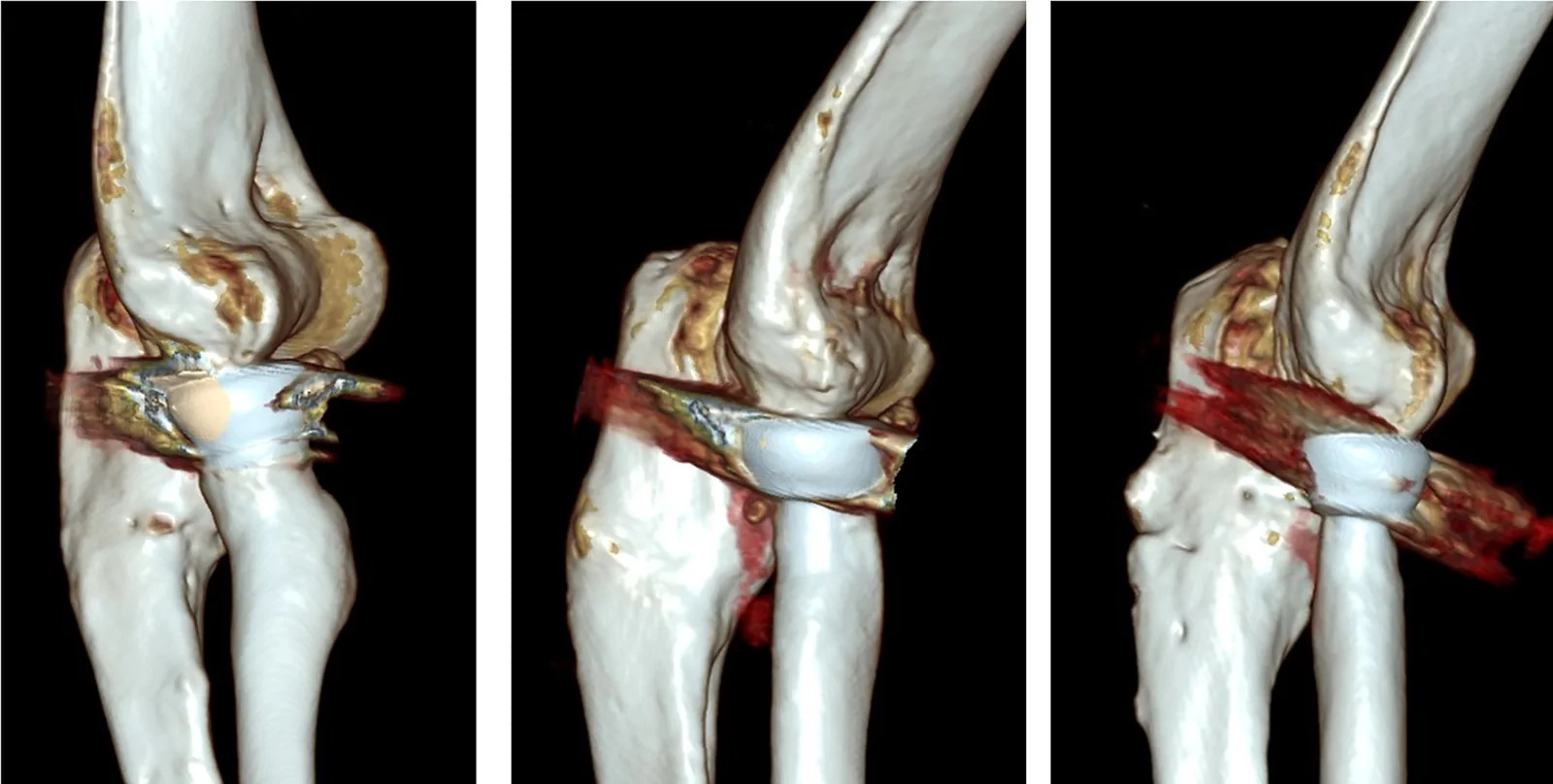
Postoperative CT showing maintained radiocapitellar reduction, prosthesis position, and ulnar osteotomy alignment.

In our preliminary clinical experience, this technique was performed in three patients with chronic Monteggia lesion and severe radial head deformity (Table 1). At short-term follow-up (minimum 16 months, maximum 26 months), radiocapitellar reduction was maintained (Figure 8), and patients reported improvement in elbow pain, wrist pain, and valgus deformity (Table 2). No evidence of prosthetic loosening, overstuffing, or loss of reduction was observed during follow-up. No nerve palsy was reported in the three patients.

**Table 1:** Patients’ demographics and pre-operative symptoms.

Case	Age/Sex	Side (Dominance)	Main symptoms	Preoperative Ext-Flex	Preoperative Pro/Sup	QuickDASH	MEPS	VAS	Osteotomy healing time
1	39/M	Right (Dominant)	Forearm pain sustained for 8 years/Instability/Deformity	0–135°	55/95°	45	60	5	7 months
2	31/M	Right (Dominant)	Forearm pain sustained for 1 year/Instability/Deformity	19–133°	54/90°	50	45	8	7 months
3	41/M	Right (Dominant)	Forearm pain sustained for 1 month/Instability/Deformity	15–130°	55/107°	27	75	4	3 months

M indicates male; Ext, extension; Flex, flexion; Pro, pronation; Sup, supination; QuickDASH, Quick Disabilities of the Arm, Shoulder, and Hand; MEPS, Mayo Elbow Performance Score; VAS, visual analog scale for pain.

**Table 2:** Clinical outcomes.

Case	Follow-up (Months)	Postoperative Ext-Flex	Postoperative Pro/Sup	Postoperative MEPS	Postoperative QuickDASH	Postoperative VAS	SOD	Complications
1	26	5–142°	38/115°	85	22	2	9	Delayed union
2	19	35–130°	17/103°	95	26	1	5	Delayed union
3	16	34–127°	38/98°	80	26	2	0	None

Ext indicates extension; Flex, flexion; Pro, pronation; Sup, supination; QuickDASH, Quick Disabilities of the Arm, Shoulder, and Hand; MEPS, Mayo Elbow Performance Score; VAS, visual analogue scale for pain; SOD, surgical outcome determination.

### Challenges and limitations

This technique has several limitations. Postoperative immobilization is often necessary to protect the osteotomy and ligament repair, but prolonged immobilization may increase the risk of elbow stiffness. This is particularly relevant in patients who have relatively preserved preoperative motion. In selected cases, secondary procedures such as plate removal and arthroscopic elbow release may be considered.

Additionally, this technique may not be appropriate for young pediatric patients because of concerns related to skeletal growth and long-term implant survival. Even in skeletally mature patients, radial head arthroplasty performed at a relatively young age raises concerns regarding long-term implant survivorship, degenerative changes, implant loosening, and recurrent joint instability. Therefore, careful patient selection, thorough preoperative counseling, and judicious consideration of the risks and benefits are essential before undertaking this salvage procedure.

Our current experience is limited to a small number of cases with short-term follow-up. Further studies with larger cohorts and longer follow-up are needed to define the ideal indications, functional outcomes, and complications of this procedure.

## Conclusion

This technical report describes a novel reconstructive strategy for the management of chronic Monteggia lesion with severe radial head deformity (Table 3). In longstanding cases, the radial head may become enlarged, flattened, or dysplastic, making stable reduction unachievable with isolated corrective ulnar osteotomy. In this subset of patients, corrective ulnar osteotomy combined with radial head replacement and annular ligament/lateral ligament complex repair may serve as a salvage reconstructive option. Although early experience is encouraging, larger studies with longer follow-up are required to determine the durability, functional outcomes, and complication profile of this approach.

**Table 3:** Key surgical steps: Pearls and pitfalls.

Surgical step	Pearls	Pitfalls
Preoperative planning	Use CT with 3D reconstruction to evaluate radial head deformity, radial notch remodeling, and ulnar deformity	Assuming that ulnar osteotomy alone will restore congruity in a severely deformed radial head
Exposure	Boyd approach allows access to both the radial head and proximal ulna through one incision	Excessive anterior radial neck dissection may increase risk to the posterior interosseous nerve
Radial head resection	Preserve radial neck bone stock and avoid excessive shortening	Over-resection may compromise stability and implant height
Ulnar osteotomy	Adjust dorsal angulation according to intraoperative radiocapitellar tracking	Treating 30° correction as a universal value; Lack of stable fixation may lead to osteotomy nonunion
Radial head replacement	Trial implant through flexion-extension and forearm rotation; avoid over-stuffing	Oversizing may restrict motion and increase capitellar wear
Contouring	Remove only impinging bone to improve smooth tracking	Excessive contouring may compromise joint support
Ligament repair	Repair annular/LUCL/capsular tissue with the radial head centered	Repairing under excessive tension may restrict rotation
Rehabilitation	Balance protection of osteotomy and ligament repair with early controlled motion	Prolonged immobilization may increase stiffness

## Data Availability

The data supporting this technical report are available on request from the corresponding author, Jiuzhou Lu, upon reasonable request.
